# Functional role of positively selected amino acid substitutions in mammalian rhodopsin evolution

**DOI:** 10.1038/srep21570

**Published:** 2016-02-11

**Authors:** Miguel A. Fernández-Sampedro, Brandon M. Invergo, Eva Ramon, Jaume Bertranpetit, Pere Garriga

**Affiliations:** 1 Grup de Biotecnologia Molecular i Industrial, Centre de Biotecnologia Molecular, Departament d’Enginyeria Química, Universitat Politècnica de Catalunya, Edifici Gaia, Rambla de Sant Nebridi 22, 08222 Terrassa, Catalonia, Spain; 2 IBE – Institute of Evolutionary Biology (CSIC-Universitat Pompeu Fabra), CEXS-UPF-PRBB, Barcelona, Catalonia, Spain

## Abstract

Visual rhodopsins are membrane proteins that function as light photoreceptors in the vertebrate retina. Specific amino acids have been positively selected in visual pigments during mammal evolution, which, as products of adaptive selection, would be at the base of important functional innovations. We have analyzed the top candidates for positive selection at the specific amino acids and the corresponding reverse changes (F13M, Q225R and A346S) in order to unravel the structural and functional consequences of these important sites in rhodopsin evolution. We have constructed, expressed and immunopurified the corresponding mutated pigments and analyzed their molecular phenotypes. We find that position 13 is very important for the folding of the receptor and also for proper protein glycosylation. Position 225 appears to be important for the function of the protein affecting the G-protein activation process, and position 346 would also regulate functionality of the receptor by enhancing G-protein activation and presumably affecting protein phosphorylation by rhodopsin kinase. Our results represent a link between the evolutionary analysis, which pinpoints the specific amino acid positions in the adaptive process, and the structural and functional analysis, closer to the phenotype, making biochemical sense of specific selected genetic sequences in rhodopsin evolution.

Vision starts when light is absorbed by the visual pigments of the retinal photoreceptor cells in the eye. Rhodopsin is the visual pigment of the vertebrate retina responsible for vision at low light intensities. It consists of the seven transmembrane G-protein coupled receptor opsin and the 11-*cis*-retinal chromophore bound to the protein by a Schiff base linkage. Rhodopsin is expressed in the rod photoreceptor cells and resides in the disc membranes of the rod outer segments where it is densely packed. Upon activation by light, the protein undergoes a series of conformational changes. Light-activated rhodopsin in its metarhodopsin II (Meta II) conformational state interacts with and activates its G (from GTP-binding) protein, transducin (Gt), which subsequently initiates a second-messenger signalling pathway that ultimately results in a neural signal to the brain.

Molecular studies are needed to determine the role of specific amino acid substitutions on the structure and function of these visual proteins during their evolution^1^. One of the main phenotypic marks of visual opsins is their ability to adapt to different light environments and to detect different wavelengths of light by means of a highly specialized spectral tuning mechanism. Molecular genetic analyses of the spectral tuning began when bovine rhodopsin^2^ and human red, green, and blue opsin genes were cloned^3^,^4^. Several studies have identified putatively adaptive substitutions in opsin sequences by statistical means, relying on previous spectral tuning analyses to infer physiological relevance of these substitutions^5^,^6^,^7^. However, few studies have attempted to test directly the physiological effects of substitutions that are predicted to be adaptive. In a landmark study, ancestral opsin sequences were reconstructed in order to determine the key, adaptive substitutions affecting wavelength shifts in spectral tuning^8^. They then performed a *post hoc* statistical analysis to test for positive selection (that is, adaptive natural selection that results in the fixation of an advantageous substitution) and found that none of the statistically predicted sites matched their experimentally determined sites. These results would indicate an uncoupling between positions relevant for function and positions showing evidence of positive adaptive selection; however, the model of positive selection used in the analysis assumes that positive selection occurred pervasively throughout the phylogeny, while it is likely that a more appropriate model would have been one in which selection is assumed to have occurred episodically, at specific points during phylogenetic divergence. This is the more general model accepted today in molecular evolution.

The visual pigments of vertebrates evolved about 500 million years ago with four spectrally distinct classes of cone opsins which appeared to have evolved through gene duplication. Rod opsin, the dim-light photoreceptor, was the result of gene duplication of the green cone opsin. Gene duplication has resulted in a high number of opsins as a result of opsin molecular evolution^9^.

The ancestor visual pigment complex in *Tetrapoda* was composed by 4 cone opsins (SWS1, SWS2, LWS and Rh2) and one rhodopsin (Rh1) for the dim and nocturnal light. Some amino acid residues in rhodopsin appear to have been positively selected during, in particular, mammalian divergence. This strong positive selection detected mainly in the *Theria* branch (live-bearing mammals, excluding monetremes such as the platypus; see Fig. 1) could be related to the loss of Rh2 and SWS2 in this lineage. Thus, the *Theria* ancestors were able to absorb just blue/UV (SWS1), red (LWS) and dim light (rhodopsin)^10^. This evolutionary loss of visual pigments probably put rhodopsin under selective pressure to compensate for the lost cone opsins’ functionalities. In primates and other mammalians, due to a subsequent LWS gene duplication, a new pigment, MWS, termed green cone opsin appeared, restoring trichromatic vision in these species^10^. It has been proposed that shared residues, between monotreme on the one side and reptile and amphibian rhodopsins on the other side, include amino acids 7, 8, 13, 225, 346 and 348 (the numbering of the amino acids corresponds to bovine rhodopsin)^11^. In the present study, we have confirmed and refined these statistical predictions stressing the main strength of selection at positions 13, 225 and 346. These three positions would then be fundamental in the adaptive process of eutherians (nowadays placental mammals). To experimentally test this, we have mutated these sites (F13M, Q225R and A346S to obtain the ancestral M13, R225 and S346), one at a time, in the background of the bovine opsin gene, and characterized them to unravel the structural and functional features of these changes as accounting for their role as positively selected sites in rhodopsin evolution, and thus at the base of main adaptive processes.

We find that the amino acid at position 13 is involved in folding of the receptor and in proper protein glycosylation. The amino acid at position 225 appears to play a key role in the function of the protein affecting the G-protein activation process, and amino acid at position 346 would also regulate functionality of the receptor by enhancing G-protein activation and could also alter rhodopsin trafficking^12^. These findings underscore the importance of molecular analysis of putative positively selected sites in rhodopsin evolution and the relevance of structural and functional analysis of these amino acid substitutions to decipher the molecular clues governing visual pigment evolution.

## Results

### Rhodopsin molecular evolution

When estimating rhodopsin’s evolutionary rate via the simple M0 model, in which a single dN/dS ratio is estimated for the entire sequence (see Materials and Methods), the sequence was found to have evolved, as expected, predominantly under strong purifying selection (dN/dS = 0.045, log-likelihood (lnL) = −12788). Thus, in general, novel non-synonymous nucleotide substitutions in this gene tend to be removed by natural selection. However, positive selection is expected to occur at individual, adaptive sites, which the M0 model cannot be used to detect. We thus performed two tests of positive selection using site-substitution models, which allow each site in the sequence to have evolved at an independent rate. Positive selection is inferred by comparing two nested models in which the alternative model includes sites with dN/dS greater than 1, and significance is determined via a likelihood-ratio test with the p-value derived from the χ^2^ distribution. These tests, if significant, would largely provide evidence of sites that have undergone positive selection throughout the phylogenetic divergence. Both tests of pervasive positive selection failed to produce significant evidence (models M1a vs. M2a: M1a log-likelihood (lnL) = −12672, M2a lnL = −12672, degrees of freedom (d.f.) = 2, p = 1; models M7 vs M8: M7 lnL = −12364, M8 lnL = −12364, d.f. = 2, p = 1). Clearly, results of pervasive positive selection should not be used and the detailed analysis has to concentrate in very specific positions within the protein/DNA sequence.

**Figure 1:**
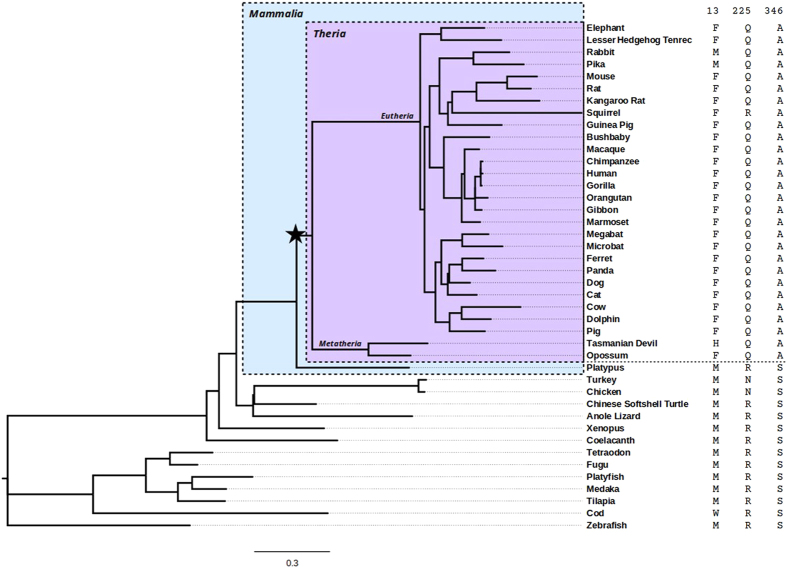
Rhodopsin phylogenetic tree. The topology of the tree, including branch lengths, is derived from the species tree computed for Ensembl Compara. The star indicates the base of the therian branch, on which we found evidence of episodic positive selection. The amino acids present at bovine positions 13, 225 and 346 are shown on the right for each species. Species above the dashed line belong to the therian clade.

We next tested the hypothesis that rhodopsin underwent episodic positive selection specifically during therian divergence via a test using a branch-site model (Fig. 1). This test allows for sites to have undergone positive selection on a specific branch of the phylogeny and significance is determined similarly to the previous tests. Rhodopsin was found to have significant evidence of positive selection on the branch leading to the therian mammals (null lnL = −12734, alternative lnL = −12731, d.f. = 1, p = 0.014). The three sites identified as having the highest posterior probability of having been targets of positive selection were (bovine coordinates and posterior probabilities given): M13F (Prob = 0.995), R225Q (Prob = 0.982) and S346A (Prob = 0.888). These three positions (the first two with strong statistical significance) clearly acquired some relevant function that resulted in being positively selected in the basal branch leading to therian mammals.

### Electrophoretic and spectroscopic characterization of the rhodopsin substitutions

**Figure 2:**
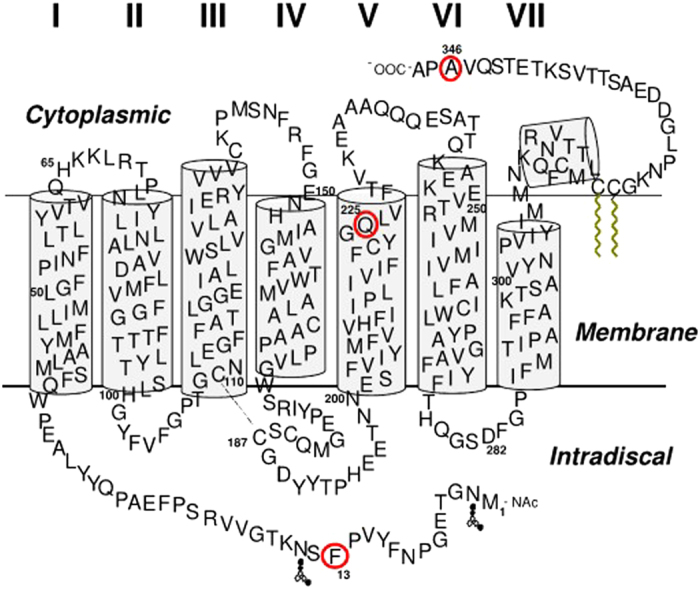
Rhodopsin secondary structure model. Sites mutated in the present study, 13, 225 and 346 are circled in red.

These three specific sites (13, 225, and 346 in the bovine opsin background), determined by the statistical analysis, were chosen for experimental characterization due to their high posterior probability of being positively selected. While site 346 does not meet the canonical probability cut-off of 0.95, its location in a region known to be physiologically relevant prompted us to include it in the experimental analysis. The ancestral mutations F13M, Q225R and A346S were constructed in the bovine opsin gene by site directed mutagenesis. These amino acids are located at the intradiscal N-terminal domain (F13), the cytoplasmic end of transmembrane helix V (Q225), and the C-terminal tail of the photoreceptor protein opsin (A346), respectively (Fig. 2).

**Figure 3:**
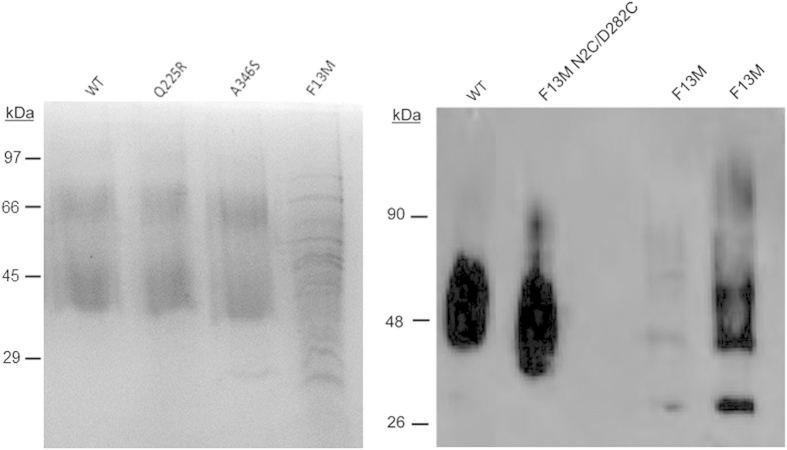
SDS-PAGE and Western blot of immunopurifed WT and mutants rhodopsins. ***Left panel***. ROS (rhodopsin from rod outer segments), WT rhodopsin and Q225R, A346S and F13M mutants are indicated in the corresponding lanes. All the mutants show a similar electrophoretic behaviour as the WT except for F13M which shows an altered pattern consistent with altered glycosylation. ***Right-panel.*** Western blot of immunopurified protein samples detected with Rho-1D4 monoclonal antibody. WT rhodopsin, F13M in the N2C/D282C background and F13M rhodopsin. Note that a 28 kDa band is clearly detectable in the F13M mutant lane.

We used electrophoretic analysis in order to determine the glycosylation and oligomerization state of the mutants which are important structural determinants of their functionality. To this end, the recombinant mutated proteins, F13M, Q225R and A346S, were expressed in COS-1 cells, immunopurified and subsequently analyzed by SDS-PAGE. The electrophoretic pattern of Q225R and A346S mutants was very similar to that of the WT (Fig. 3, left panel) showing the characteristic trailing smear typical of rhodopsin expressed in COS-1 cells and attributed to heterogeneous glycosylation^13^. However, the F13M mutant showed a clearly altered pattern, with a series of discrete bands and the appearance of lower bands below the opsin main band (at about 40 KDa) that can be attributed to non-glycosylated^14^ or truncated opsin species^15^.

**Figure 4:**
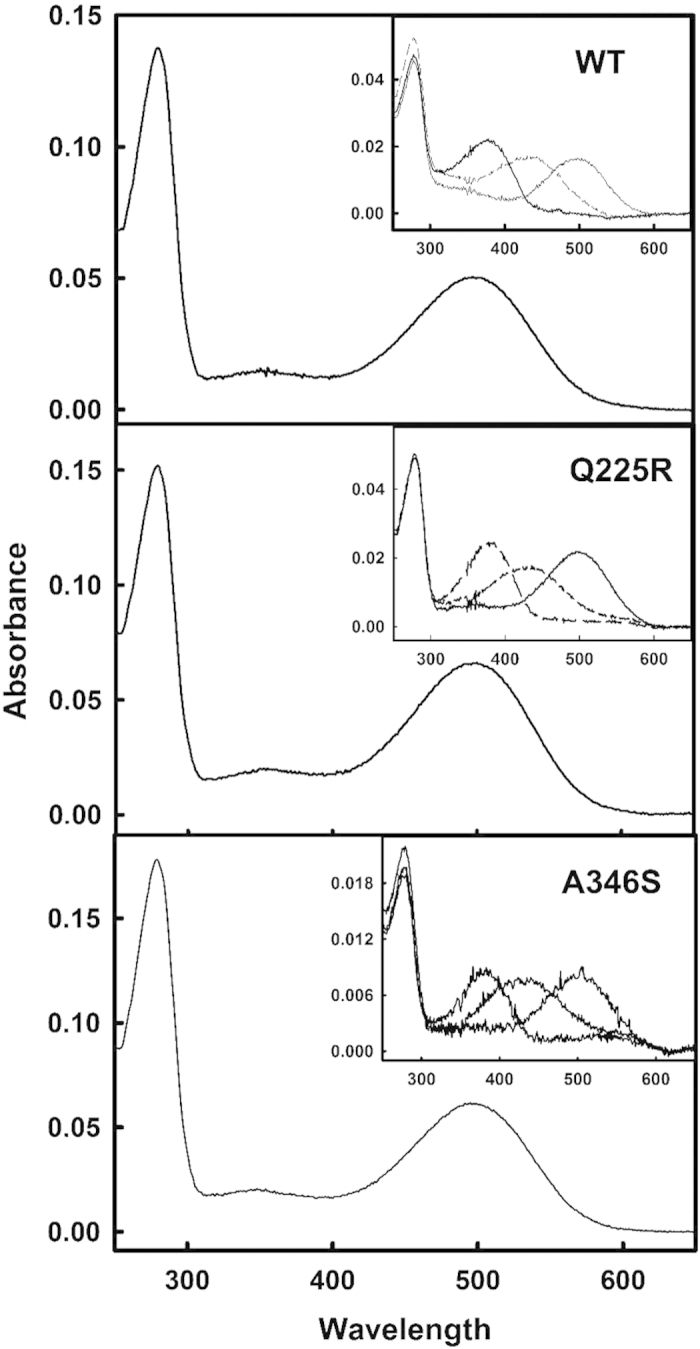
Spectroscopic UV-vis characterization of the purified proteins. UV-vis spectra of WT, Q225R and A346S in dark. Insets show the corresponding dark (λ_max_ = 498 nm), photobleached (λ_max_ = 380 nm), and acidified spectra (λ_max_ = 440 nm). Note that the mutants show photobleaching and acidification behaviours analogous to those of WT rhodopsin.

**Figure 5:**
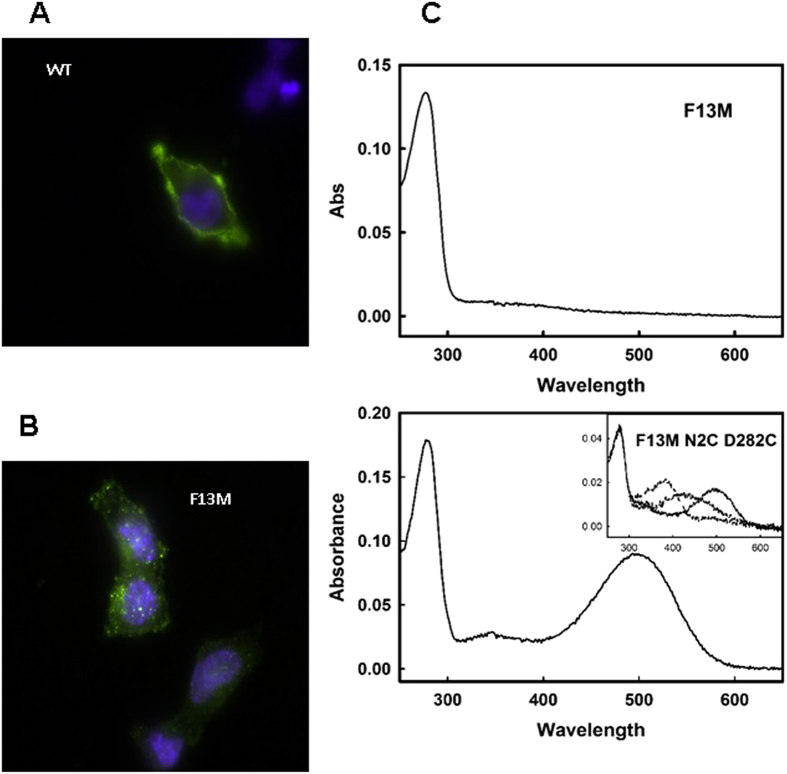
Fluorescence microscopy and UV-vis spectroscopy behaviour of F13M mutant. Transfected cells with WT rhodopsin **(A)** and F13M mutant **(B)** were analyzed by fluorescence microscopy. The blue colour corresponds with the nucleus of the cells, and opsins are labelled in green. (**C**) UV-vis absorption spectra of F13M in the dark (top panel) showing no chromophore regeneration in the visible region. When the mutation is obtained in the background of the N2C/D282C double mutant, then chromophore regeneration can be rescued to WT levels (lower panel). *Inset*, photobleaching and acidification spectra of the rescued mutant.

One of the main adaptively relevant properties of rhodopsins is their light absorption capacity. Thus, the spectral behaviour of the purified proteins was analyzed by UV-vis spectroscopy and its light absorption properties were determined in their dark-adapted state (Figs 4 and 5C). Wild-type (WT) rhodopsin showed the characteristic visible band at 500 nm and the mutants Q225R and A346S showed visible bands at the same wavelength (Fig. 4). These two mutants showed similar levels of chromophore regeneration with retinal than WT rhodopsin as judged by their A_280_ _nm_/A_500_ _nm_ ratios (see Table 1). The photobleaching and acidification spectra were determined immediately after illumination (with light of λ > 495 nm) and after acidification respectively. Upon illumination, both mutants showed a typical absorbance band at 380 nm, corresponding to the active Meta II conformation. Subsequent acidification of the samples shifted the absorption maximum from 380 nm to 440 nm which corresponds to the reprotonation of the Schiff base nitrogen. Thus, we find a WT-like behaviour for the Q225R and A346S mutants in the photobleaching and acidification assays (Fig. 4, insert), suggesting that these amino acid changes did not alter the pathway of photointermediates leading to the activated receptor.

A specific behaviour was observed in the case of the F13M mutation, at the N-terminal domain of the receptor, which did not show detectable chromophore regeneration as detected by the lack of absorbance in the visible region (Fig. 5C, upper panel). This lack of chromophore regeneration ability could reflect protein misfolding. It is known that misfolded opsins are retained in the endoplasmic reticulum or can form intracellular inclusions due to a failure in the intracellular transport to the plasma membrane. Thus, we analyzed the subcellular localization of the F13M mutant, expressed in COS-1 cells, and compared it to that of WT rhodopsin in order to confirm structural misfolding of this mutant. A clearly different pattern was observed in the two cases with WT opsin being trafficked to the plasma membrane (Fig. 5A), whereas F13M appeared not to be localized effectively to the plasma membrane, and formed intracellular inclusions with higher frequency, in a pattern consistent with protein misfolding (Fig. 5B).

### Rescue of chromophore regeneration for the F13M mutant

It was of interest to find out if the misfolded phenotype for the F13M could be rescued by means of an experimental strategy. Therefore, pharmacological chaperone rescue was carried out with the F13M mutant. For this, COS-1 cells transfected with this mutant gene where incubated in the presence of 9-*cis*-retinal. Previous studies showed that defective N-terminal mutants supplied with 11-*cis*-retinal or 9-*cis*-retinal, during protein biosynthesis, could recover WT-like rhodopsin chromophore regeneration levels^16^,^17^. In the case of F13M we could not get any detectable chromophore regeneration for this mutant using this strategy (supplementary Figure S1).

We hypothesized that the inability of the F13M mutant to bind retinal was due to the fact that this mutation, at the N-terminal domain of the receptor, could be destabilizing the protein conformation, thus affecting receptor folding and altering at the same time glycosylation at the proximal N15 residue^18^. In order to stabilize the structure, we introduced the F13M mutation in the background of the N2C/D282C double mutant that forms a disulphide bond between Cys2 and Cys282 increasing opsin stability^19^. By using this strategy we could recover full chromophore regeneration for F13M mutant to a similar extent to that of WT rhodopsin (Fig. 5C, lower panel). Furthermore, Western blot analysis of F13M showed a clear distinctive lower band at approximately 28 KDa that was not detected when the mutant was obtained in the Cys2/Cys282 background (Fig. 3 right panel). In this latter case a pattern similar to that of WT could be observed, consistent with the rescue observed for chromophore regeneration.

In order to rule out that the retinal could be binding to other Lys residues^20^ in the F13M mutant (other than the natural K296 at transmembrane helix 7) we constructed the quadruple mutant F13M/N2C/D282C/K296G where the site of retinal attachment was eliminated by the K296G mutation^21^. We could not get any chromophore formation for this mutant indicating that retinal was binding to the native K296 in the rescued triple mutant (Supplementary Figure S2).

### Conformational stability and functionality of WT and mutant opsins

One of the important aspects underlying the functionality of rhodopsin in visual perception is the structural stability of both the dark-adapted and the illuminated photoactivated states. Specific amino acid substitutions can have a profound impact on the stability of the protein so it is of interest to determine their thermal and chemical stabilities in the dark and also the stability of the activated Meta II state. The influence of the mutations on the specific function, i.e. G-protein activation, is also a relevant parameter that can shed light on the importance of a given amino acid position in the molecular evolution of the protein.

#### Dark-state chemical stability

**Figure 6:**
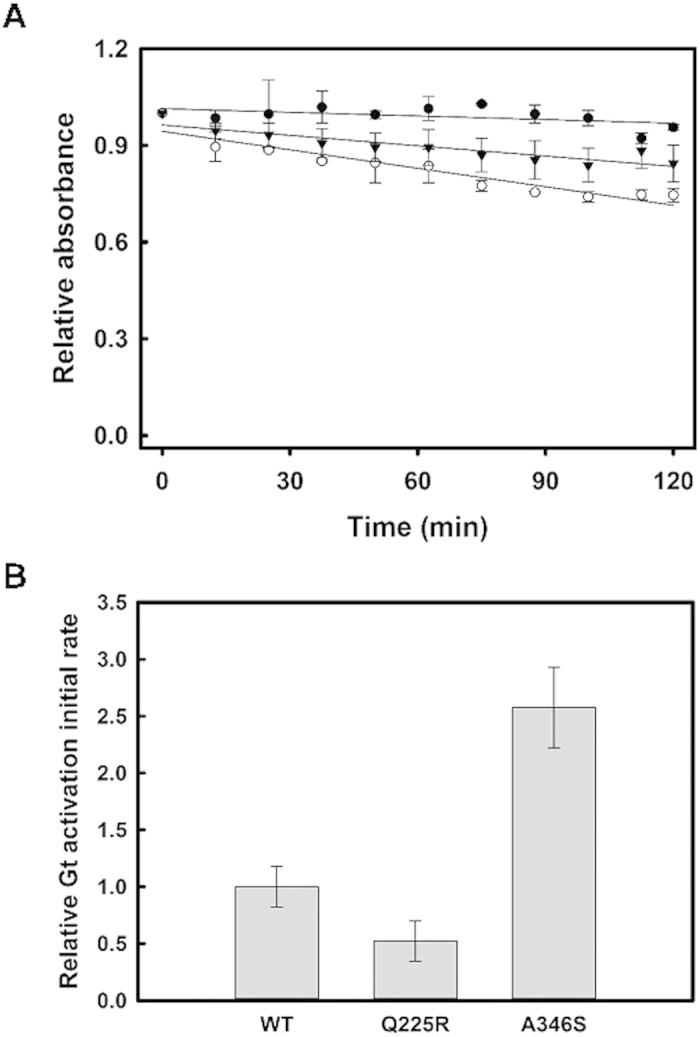
Molecular properties of immunopurified mutants. (**A**) Chemical stability in the presence of 50 mM hydroxylamine. The decrease in absorbance at the visible λ_max_ was measured over time. WT rhodopsin (●), Q225R (▾) and A346S (○) (**B**). Relative Gt activation initial rate. Error bars represent S.E. in both panels.

We determined the hydroxylamine reactivity of WT and the mutants in the dark state. Hydroxylamine cannot access the compact WT rhodopsin binding pocket in the dark state but if the conformation becomes more open, as in the case of mutation, then it can enter the binding pocket forming a retinal oxime with 11-*cis*-retinal^22^. Thus, hydroxylamine is used to measure the chemical stability of rhodopsin in the dark as an indirect measure of the accessibility of the Schiff base linkage under these conditions. WT has a high stability towards hydroxylamine in the dark (Table 1) indicating that the retinal Schiff base is not accessible under these conditions. Both Q225R and A346S mutants show a slightly increased sensitivity towards hydroxylamine in the dark (Fig. 6A) that would reflect a less compact structure around the Schiff base linkage in the chromophore binding pocket.

#### Dark-state thermal stability

Another assay used to assess the stability in the dark state, is to follow the decay of the visible band at 48 °C. At this temperature the Q225R mutant showed similar thermal bleaching kinetics as the rhodopsin WT, while the A346S mutant showed slightly faster bleaching kinetics (Table 1). On the other hand, the mutant F13M/N2C/D282C has a high stability as expected from the stabilizing effect of the additional engineered disulfide bond (t_1/2_ > 120 min at this temperature) as previously described^23^.

#### Meta II stability

Meta II decay was determined, in real time, by monitoring Trp fluorescence increase upon rhodopsin photoactivation. Our data showed a similar decay time for the Q225R and A346S mutants when compared to WT (Table 1).

## Functional Gt activation. 

We analyzed the functionality of the mutant rhodopsins by means of a radioactive assay that measures the ability of the mutant rhodopsins to activate the Gt. We found a lower initial rate for Gt activation for the Q225R mutant compared with the WT. Conversely, A346S mutant showed an increase in Gt activation rate (Fig. 6B, Table 1).

## Discussion

The functional variability among vertebrate rhodopsins is not well studied, except for spectral tuning, and little is known about other phenotypic features like glycosylation, G-protein activation and phosphorylation. In this regard, most of the RH1 pigments have λ_max_ values at about 500 nm except for some fishes that have a 10-20 blue-shifted λ_max_ that could reflect an adaptation to the ocean environments^1^. Some variability has also been found in the kinetics of rhodopsin photointermediates, like the Meta II decay rates that are significantly different in human and bovine rhodopsin compared to other non-mammalian rhodopsins^24^. Finally, mathematical models of amphibian^25^ and mammalian^26^ phototransduction suggest that the binding between rhodopsin and Gt and the activation of the G protein occur at significantly faster rates in mammals. All the existing information points towards the existence of functional differences in the evolution of vertebrate rhodopsins.

Previous studies suggested that certain specific amino acid positions could be important for vertebrate rhodopsin evolution^11^. We have extended these previous analyses by using a larger phylogenetic tree and more opsin sequences that make our analysis more reliable and trustworthy for deep functional analyses. We find that the sites F13, Q225 and A346 are the three sites with the highest posterior probability of having been targets of positive selection after mutating from their ancestral states (M13 → F13, R225 → Q225 and S346 → A346) during the evolution of mammals. Based on our statistical analysis we have constructed, expressed and immunopurified these three reverse mutations in the bovine opsin background and we have analyzed in detail the structural and functional properties of the purified mutants. We find a clear structural alteration for F13M mutant and stability and functional alterations for the other two mutations, Q225R and A346S that can have importance in the protein molecular evolution process.

**Figure 7:**
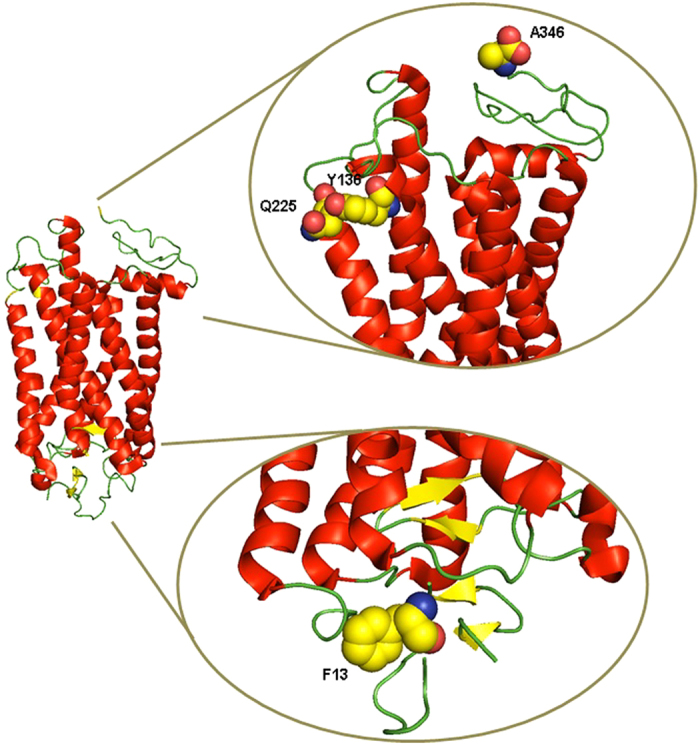
Three dimensional model of the inactive structure of rhodopsin. The three amino acids studied (positions 13, 225 and 346) are highlighted in yellow. Glu225 is found to be interacting with Tyr136 of the highly conserved Glu134-Arg135-Tyr136 motif which plays a critical role in rhodopsin activation.

Gln225 is located at the cytoplasmic end of transmembrane helix V in the junction region with the third cytoplasmic loop. Previous studies have shown that this loop is directly involved in Gt binding and activation that occurs after rhodopsin photoactivation^27^. A different substitution at this position, Q225C was studied in a cysteine-scanning mutagenesis approach where all the amino acids at or near the third cytoplasmic loop were mutated to Cys^28^. In that case, mutation to Cys did not affect rhodopsin folding but on the contrary it resulted in slightly increased Gt initial activation rate^28^. In our case, the substitution to an Arg in the Q225R mutation would introduce a bulky, charged residue in a domain that is critical for Gt activation. This Arg would likely steric clash with Tyr136 of neighbouring helix III (Fig. 7) that belongs to the conserved ERY motif critical in Gt activation. Arg135 in this conserved triplet was shown to be hydrogen bonded with a Gt-derived peptide^29^,^30^. Any change in the structure of this region would cause a change in the coupling efficiency to the G-protein and could explain the decreased Gt activation observed for Q225R when compared to WT rhodopsin. Recent studies indicate that the finger loop in arrestin interacts in a similar way with Arg135^31^ so the Q225R mutation could also potentially affect arrestin binding but this would require further investigation.

In contrast, A346S, the other studied mutation that regenerated chromophore, showed increased Gt activation rate (Fig. 6B). Ala346 is the site of the naturally-occurring A346P mutation associated with the retinal degenerative disease retinitis pigmentosa (RP)^32^,^33^ that was studied together with other disease causing mutants^34^. In that study some mutants associated with RP were found to have higher Gt activation rates and this was proposed to be the cause of their disease phenotype. Also, other studies demonstrated that some mutations in the C-terminal affect the *in vivo* trafficking of rhodopsin, resulting in random delivery to photoreceptor cell membranes^12^,^35^. Although the C-terminal tail of rhodopsin is believed to be devoid of a defined structure and quite flexible^36^ we can propose that the C-terminal tail may be covering part of the third cytoplasmic loop in the dark state that would regulate the Gt activation process. In the case of mutations at this domain, these amino acid changes could affect the conformation of the C-terminal tail of rhodopsin allowing exposure of the third cytoplasmic loop and facilitating the rhodopsin-G-protein recognition process. Moreover, the specific A346S mutation creates a potential additional phosphorylation site in the protein which would affect rhodopsin phosphorylation after photoactivation^37^ and in turn could affect the binding affinity of arrestin, a regulator of rhodopsin deactivation. This could result in a reduction of the amplitude and duration of the flash response since dephosphorylation of rhodopsin correlates with dark adaptation^38^,^39^. Interestingly, it has been recently shown that mutation of Ser to Ala at the C-terminal tail in rhodopsin of transgenic mice results in slowed-down response kinetics^40^. Therefore, this extra phosphorylation site on the ancestral pigment could provide an evolutionary explanation for the enhanced response in the case of Gt activation that would need a more efficient signal desensitization process.

Both the Q225R and the A346S electrophoretic and spectroscopic patterns were similar to those of WT rhodopsin indicating that both mutants are preferentially in a monomeric state and that the mutations do not alter their UV-vis spectral properties. Thus, the WT-like behaviour for the Q225R and A346S mutants suggests that they did not alter the pathway of photointermediates leading to the activated receptor. The same WT-like behaviour was found in the Meta II decay for both mutants and in the thermal stability for the Q225R rhodopsin, whereas the A346S rhodopsin showed only slightly decreased thermal stability when compared to WT (Table 1). This minor difference is not believed to play a significant role in function, and thus in rhodopsin evolution. In contrast, we found a higher sensitivity to hydroxylamine than WT for both Q225R and A346S mutants (Fig. 6A). This would reflect a less compact structure for the mutants in the Schiff Base linkage environment; the sensitivity to hydroxylamine has been used in previous studies to distinguish rod opsins from cone opsins, since this reaction is substantially faster in cone opsins^22^. Other studies in echidna rhodopsins (the most basal mammals) revealed that these rhodopsins are significantly more sensitive to hydroxylamine than bovine rhodopsin^10^. Thus, the observed increased response to hydroxylamine in the dark state would suggest a phenotype somehow closer to cone opsins for the ancestral state. Cone pigments would require more flexible and conformationally adjustable binding pockets to allow efficient retinal binding for a fast physiological response as observed when comparing cone pigments and rhodopsin ligand binding properties^41^. Therefore, the physiological correlate of these results could be found in the known difference between cone and rod opsins that is related with the life-time of their signalling states. Cone opsins can form their signalling states so that they can respond faster (and have a faster dark adaptation) in mediating color and diurnal vision, but they would actually do so by compromising their sensitivity in comparison to the more sensitive rod opsin. In contrast, rod photoreceptors have a much longer signalling state to account for an amplified response that increases the sensitivity of the photoreceptor, slowing down the physiological response, regulating dark adaptation and making scotopic vision possible^42^.

In the case of the F13M, the poor expression observed is not likely related to problems with trafficking in COS cells because we have also obtained the same results in HEK293 cells. Different vertebrate rhodopsins from species that have M at position 13 have been successfully expressed in HEK293 cells (i.e. chicken^43^ and zebrafish^24^) or in COS cells (i.e. zebrafish^44^ and Astyanax^45^). In the case of the Astyanax fish, the A_280_/A_500_ ratio of the purified pigment was reported to be much higher than that of bovine rhodopsin, and the SDS-PAGE pattern indicated apparent differences in the glycosylation pattern^45^. Therefore, we believe that expressing the protein either in COS cells or in HEK293 cells does not affect trafficking although it can alter the glycosylation pattern of the expressed protein.

In our case, we find that the F13M mutation causes misfolding and alters glycosylation of the protein in spite of the relative conservation of the substitution. Phe13 is near the position of the glycosylation site located at Asn15 (Fig. 2). Some mutations near this position, like N15S and T17M, are linked to a subset of the retinal degenerative disease RP, known as sector RP, in which the inferior retina appears to be primarily affected^46^,^47^,^48^. There is another glycosylation position at Asn2. Human disease and animal models suggest that glycosylation plays an important role in the structure and/or function of rhodopsins^17^,^46^. In recent years, the importance of the N-terminus in rhodopsin has been emphasized and the importance of including the N-terminus and extracellular loops in structural studies has been highlighted^49^. Some studies suggest that glycosylation at Asn15 is essential for rhodopsin structure and/or function^50^, whereas glycosylation at Asn2 had no significant effect on rod viability^46^. A prominent electrophoretic 28-kDa band was observed for F13M (Fig. 3). In previous studies, a similar band has been observed in cell culture and transgenic frog or mouse models of P23H rhodopsin (associated with RP) and other mutations located in the N-terminal in bovine opsin^15^. It was thought to correspond to an N-terminal-truncated product of opsin, as it was recognized by a C-terminal antibody but lacked epitopes to N-terminal antibodies and was not glycosylated. Thus, the 28-kDa protein has been assigned to an opsin truncation product^15^.

In view that the F13M mutation causes protein misfolding and lack of chromophore regeneration we successfully attempted to rescue the folded (chromophore binding) phenotype. The results of the spectral characterization of the immunopurified mutant F13M in the N2C/D282C background showed that the mutation phenotype can be rescued in the double cysteine mutant and that in this case it can regenerate chromophore to WT levels. The photobleaching and acidification behaviour of the rescued F13M mutant are also similar to those of WT rhodopsin indicating that the rescued protein is properly folded and has a normal phototransduction process (Fig. 5C). Moreover, according to a packing analysis using the method of occluded surfaces, Phe13 residue would have van der Waals interactions with the extracellular loop 3, where Cys282 is located^17^. We could also find differences in the cellular localization in the case of the F13M mutant that formed intracellular inclusions and was not transported efficiently to the plasma membrane (Fig. 5B). This behaviour was consistent with the known endoplasmic reticulum export efficiency changes that are typically associated with misfolding of membrane proteins in heterologous expression systems^51^.

In conclusion, we find physiological relevance for specific mutations in rhodopsin derived from statistical evidence of positive selection. We show that position 13 in rhodopsin is very important for the folding of the receptor and also for proper protein glycosylation. Given the deleterious nature of the reverse substitution and our ability to recover a WT phenotype for this mutant, we speculate that the positive selection observed at this site could have been compensatory. In this case, concerted amino acid changes can be related to the fine-tuned network of amino acid interactions that govern the stability and folding of the tightly packed rhodopsin extracellular domain, and particularly its N-terminal tail. It is interesting to note that, among therians, the rabbit (*Oryctolagus cuniculus*) and the pika (*Ochotona princeps*) both show a reversion to methionine at position 13 (Fig. 1), making them potential candidates for further investigation of the role of this position. On the other side, positions 225 and 346 appear to be important for the protein functionality in the G-protein activation process and are likely sites of adaptation. Our results highlight the importance of molecular investigations of positive selected sites in rhodopsin evolution and the relevance of structural and functional analysis of these sites in unravelling the molecular basis of visual pigment evolution. This is also highlighted in a recent experimental study that provides further support to our mutational analysis particularly taking into account that nowadays some evolutionary hypotheses on vision are still based on indirect anatomical inference from fossils^52^. Overall, these findings provide a deeper insight into specific amino acids involved in rhodopsin molecular evolution and they may, at the same time, have implications for rhodopsin molecular evolution theories of early mammalian nocturnality, where these mammals would have changed their visual patterns, during the Mesozoic era, in order to avoid competition with diurnal reptiles^53^. Our results are relevant to recent studies suggesting that mechanisms of epistatic interactions between specific amino acids must be understood by studying various orthologues in different species that have adapted to different environments^54^. These findings contribute to the emerging trend that complex non-linear interactions seem to be at the base of phototransduction evolution^55^.

## Materials and Methods

### Positive selected amino acids determination

The GeneTree alignment for the human RHO gene was fetched from Ensembl Compara (version 72^56^). The alignment was filtered to include high-quality orthologous sequences from 42 vertebrate species, including paralogs in one-to-many orthologous relationships, resulting in an alignment of 48 sequences. In order to filter out divergent paralogs, which may have evolved under different selective pressures, a phylogenetic gene tree was produced from this alignment using PhyML (version 3.0^57^). The tree was constructed using the GTR substitution model, which produced the highest-likelihood tree out of the different models provided by the software (log-likelihood = −15174); otherwise, the software’s default parameters were used. Using this tree, we identified and pruned one rhodopsin paralog each in six fish species, resulting in a final alignment of 42 orthologues with a single ortholog per species.

The rate of evolution undergone by rhodopsin during phylogenetic divergence was first estimated using the M0 site-substitution model as implemented in the codeml program of the PAML package (version 4.7^58^) using the pruned gene tree. This model estimates the rates of non-synonymous substitutions (dN) and synonymous substitutions (dS) during divergence. Their ratio (dN/dS) may be used as an indication of the strength and direction of selection. Under the assumption that synonymous substitutions are nearly selectively neutral, when dN/dS is less than one, the site is taken to have been under purifying (negative) selection; when it is equal to one, the site is evolving neutrally; and when it is greater than one, the site has undergone adaptive (positive) selection. Because the M0 model uses a single dN/dS ratio for all sites in the sequence alignment, it is not very realistic, however it can be taken as an overall indicator of the strength of purifying selection acting on the gene^59^,^60^.

Three tests of positive selection were performed on the alignment. Evidence of pervasive positive selection during phylogenetic divergence was tested using two pairs of nested site-substitution models: M1a versus M2a^61^,^62^ and M7 versus M8^63^, as implemented in codeml. In both cases, each site in the alignment is assigned a class, which is associated with a given dN/dS ratio. In the null models (M1a, M7), all site classes have dN/dS ratios less than or equal to one (purifying selection or neutral evolution). The alternative models (M2a, M8) include a site class with dN/dS ratio greater than one (positive selection). The maximum likelihoods found by applying the null and alternative models are then compared via a likelihood ratio test with two degrees of freedom and a confidence level of 0.05; a significant test indicates that the alternative model featuring positively selected sites better fits the data. The M1a/M2a test is known to be more robust while M7/M8 is more sensitive^64^.

Evidence of episodic positive selection was inferred via the branch site A model. Under this model, episodic selection is tested on one branch of the phylogeny against a background of neutral or purifying selection on the rest of the tree. In the null model, sites are classified as either being under purifying selection or neutral evolution on all branches of the phylogeny; the alternative model additionally allows sites to be classified as having undergone positive selection on a single, specific phylogenetic branch. The alternative model was then tested for significance via a likelihood-ratio test with one degree of freedom and a 0.05 confidence level. We hypothesized that rhodopsin would have undergone significant adaptation early in mammalian divergence. Therefore, we marked the branch at the root of the therians as the “foreground” branch, for which positive selection would be tested.

Because gene tree topologies often differ from that of the species tree, the tests of positive selection were performed using the topology of the species tree available from Ensembl, pruned to include only the species used in this study, in order to accurately mark the branch of interest (Fig. 1). Branch lengths were estimated directly by codeml. The program was run with three different initial values of the parameter omega (dN/dS) (0.1, 1.0, 2.0), five times each, in order to affirm that the models converge to stable maximum likelihoods. Specific residues that have undergone positive selection were then predicted *a posteriori* via the Bayes Empirical Bayes method as implemented in codeml^62^.

### Construction of opsin mutants

We used site-directed mutagenesis (QuikChange II. Stratagene) on WT bovine opsin gene, in the pMT4 vector with no tags, as a template to construct the mutants A346S, Q225R and F13M. These mutations were introduced in the background of the double cysteine mutant N2C/D282C^19^. The following primers were used: *Forward primer A346S: CTTAGGCAGGGCTCACTTGGCTGGTCTC; Backward primer A346S: GAGACCAGCCAAGTGAGCCCTGCCTAAG; Forward primer Q225R: CGGTGAACACCAGCCGGCCATAGCAGAAG; Backward primer Q225R: CTTCTGCTATGGCCGGCTGGTGTTCACCG; Forward primer F13M: GCCCGTCTTGTTGGACATAGGAACGTAGAAG; Backward primer F13M: CTTCTACGTTCCTATGTCCAACAAGACGGGC.* Primers were synthetized by Sigma. The mutant plasmids were sequenced to verify the correct introduction of the mutations.

### Expression and purification of the mutant opsins

The mutants were expressed in mammalian COS-1 cells. Polyethylenimine (PEI) media was purchased from Polysciences and L-glutamine, foetal bovine serum (FBS) and antibiotics from Sigma. Cells were grown in a humidified incubator at 37 °C under 5% CO2 atmosphere.

The expression and purification were performed as follows. Plasmids encoding the WT or mutant rhodopsin genes were transfected into COS-1 cells plates at 85% confluence, by using 0.1 ml of PEI with 15 μg of plasmid DNA per plate. Cells were harvested 48–65 h after transfection, and pigments were reconstituted with 10 μM 11-*cis*-retinal (or 9-*cis*-retinal) in intact cells and then solubilized with 1% (w/v) dodecyl maltoside (DM) in PBS pH 7.4. The pigments were purified by immunoaffinity chromatography on 1D4-Sepharose 4B. All procedures, up to and including binding of the receptor to the immunoaffinity matrix, were performed at 4 °C, whereas subsequent washes and elutions were performed at room temperature (RT) using cold buffer. The resin was washed four times with 0.05% DM in PBS. The receptors were eluted the same buffer containing 100 μM rho-1D4 9-mer peptide (TETSQVAPA).

Experiments of pharmacological chaperone rescue were performed in COS-1 cells transfected with 15 μl of plasmid DNA in the presence of 50 μM of 9-*cis*-retinal. A second addition of 50 μM of 9-cis-retinal were performed 24 hours later and the plates where recollected after 48 hours. All the steps were carried out in the dark.

### Subcellular localization of mutant opsins

Cellular opsins localization was performed by means of immunofluorescence microscopy (Nikon Eclipse Ti). For this, cells were incubated in GnTI^−^ HEK-293 cells during 24 hours. Cells were washed three times with cold PBS and then immediately fixed with 3.5% formaldehyde for 30 min at 37ªC and consecutively the sample was blocked with 5% milk. We use a primary 1D4 mouse IgG for 1 hour, RT. Finally the sample were stained with anti-mouse IgG fluorescein-labeled (FITC) and incubated for 1 h at RT (cells were washed three times with cold TBS in each step). Cells were mounted with 2 μg/ml DAPI for nuclei counterstained.

### SDS-PAGE and Western-blot

Electrophoresis studies were carried out in order to compare the patterns of the purified mutant opsins with that of the WT receptor. For Western blot analysis, the monoclonal Rho-1D4 antibody was used as the primary antibody and an anti-mouse as secondary antibody.

### UV-vis spectrophotometric characterization of the mutants

All measurements were made on a Cary 100 Bio spectrophotometer (Varian), equipped with water-jacketed cuvette holders connected to a circulating water bath. Temperature was controlled by a Peltier accessory connected to the spectrophotometer. All spectra were recorded in the 250–650 nm range with a bandwidth of 1 mm, a response time of 0.1 s, and a scan speed of 600 nm/min.

Purified samples where photobleached with a 150 Watt power light using a 495 nm cut-off filter during 30 seconds; an absorption spectrum was immediately recorded. Acidification was performed immediately after photobleaching by the addition of 2 N H_2_SO_4_ to a final sample pH of 1–2 and the acidified spectrum was measured after 1 min.

### Thermal and chemical stability of the opsin mutants

Rhodopsin thermal stability in the dark was studied with the spectrophotometer at 48 °C by monitoring the rate of A500 loss, and the increase of A380 as a function of time. Spectra were recorded each 2.5 min. The chemical stability was measured by adding hydroxylamine (NH_2_OH) to the sample and monitoring the decrease in the 500 nm absorbance band with time at 20 °C. NH_2_OH was added to the sample to a final concentration of 50 mM and successive spectra were recorded every 2.5 min to measure the loss of visible absorbance (A_500_) and formation of retinal oxime (A_365_). Samples were kept in the dark until the end of the experiment when they were completely photobleached.

### Meta II decay determined by fluorescence spectroscopy

Meta II decay was recorded by fluorescence spectroscopy at 20 °C. All fluorescence spectra were carried out by exciting the samples for 2 s at 295 nm, with a bandwidth slit of 0.5 nm and measuring Trp emission at 330 nm^65^. Rhodopsin in PBS 0.05% DM was kept at 20 °C until scan was stable and illuminated for 30 s. The t_1⁄2_ values for the Trp fluorescence increase (resulting from retinal release from the receptor) were derived from fitting the obtained curves with an exponential function.

### Gt activation

Gt activation was measured with purified proteins expressed in COS-1 cells, and the ability of opsin and rhodopsin to activate Gt was monitored by means of a radionucleotide filter binding assay by measuring the uptake of [^35^ S]GTPγS by Gt purified from bovine retinas. The assays were performed by mixing 6 nM mutant rhodopsin or WT rhodopsin with 500 nM Gt in 25 mM Tris, pH 7.5, 100 mM NaCl, 5 mM magnesium acetate, 5% glycerol, 2.5 mM DTT, and 3 μM [^35^ S]GTPγS (0.128 Ci/mmol) at RT. Reactions were initiated by the addition of rhodopsin in the dark, and samples were filtrated after different incubation times, either in the dark or after illumination, to determine the amount of bound [^35^S]GTPγS^66^.

## Additional Information

**How to cite this article**: Fernández-Sampedro, M. A. *et al.* Functional role of positively selected amino acid substitutions in mammalian rhodopsin evolution. *Sci. Rep.* **6**, 21570; doi: 10.1038/srep21570 (2016).

## Supplementary Material

Supplementary Information

## Figures and Tables

**Table 1:** Spectroscopic properties of WT and rhodopsin mutants.

	WT	Q225R	A346S
λmax (dark) (mm)	498 (11-*cis-*retinal)	498 (11-*cis-*retinal)	498 (11-*cis-*retinal)
Ratio 280 nm/ λmax	2.66 ± 0.43	2.43 ± 0.19	2.75 ± 0.58
aThermal Stability (48 °C) (min)	27.8 ± 6.8	25.9 ± 4.9	19.3 ± 4.2
Meta II Decay (min)	13.9 ± 0.8	13.2 ± 1	14.5 ± 2.7
bHydroxylamine, 50 mM, Initial velocity (min^−1^)	0.000379 (1.00)	0.001068 (2.82)	0.001910 (5.04)
cGt activation initial relative rate	1.00 ± 0.179	0.52 ± 0.177	2.58 ± 0.35

^a^t_1/2_ of the thermal stability measurements of purified samples at 48 °C measured at the visible λ_max_.

^b^Chemical stability measured as the decrease rate of the absorbance at the λ_max_ absorbance in the dark in the presence of 50 mM hydroxylamine. Relative values are shown between brackets.

^c^Relative initial rates of Gt activation.
