# Supplementary material for: Functional role of positively selected amino acid substitutions in mammalian rhodopsin evolution
Source: Sci Rep. 2016 Feb 11;6:21570. doi: 10.1038/srep21570 (PMC4749998; doi:10.1038/srep21570)
Supplement: Supplementary Information [file srep21570-s1.doc]

**Supplementary information**

**Functional role of positively selected amino acid substitutions in mammalian rhodopsin evolution**

Miguel A. Fernández-Sampedro, Brandon M. Invergo, Eva Ramon, Jaume Bertranpetit and Pere Garriga


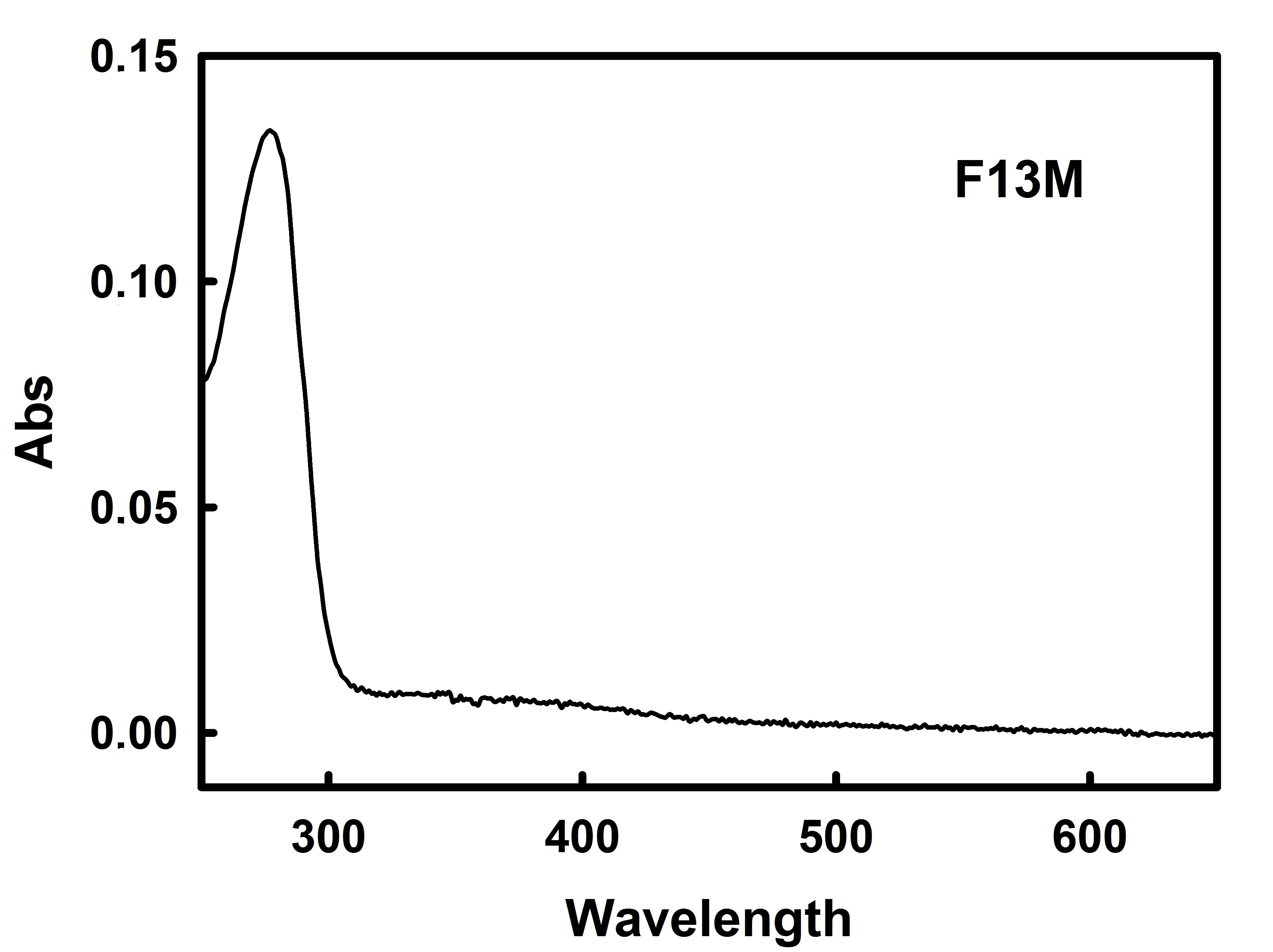


Figure S1. *UV-visible spectrum of purified F13M mutant.* The mutant was expressed in COS-1 cells and 9-*cis*-retinal was added to the cell culture during protein expression. No detectable chromophore regeneration can be observed at the 500-nm visible region.


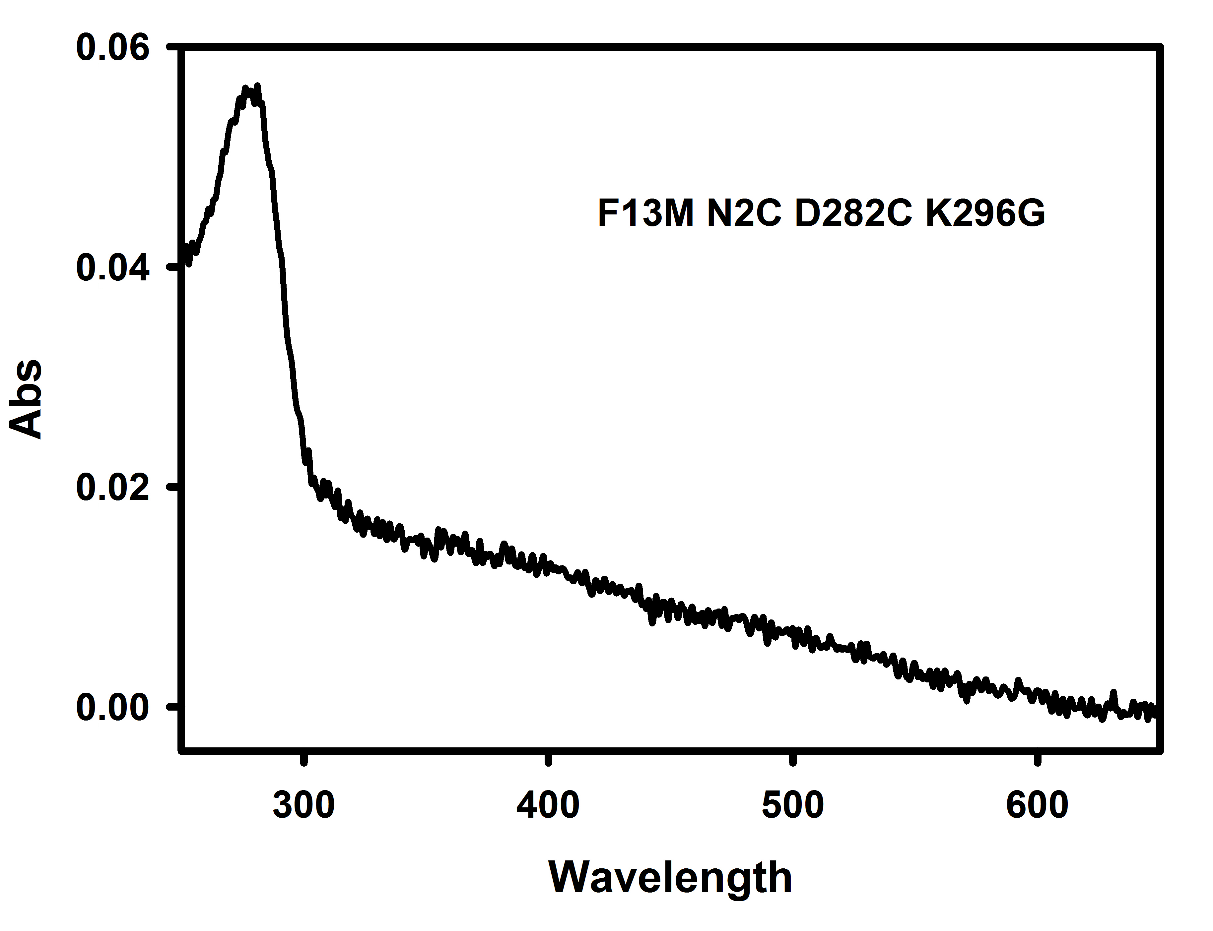


Figure S2. *UV-visible spectrum of the purified quadruple F13N/N2C/D282C/K296G mutant*. No detectable chromophore at the visible region could be observed indicating that 11-*cis*-retinal was binding to the native K296 in the triple F13M/N2C/D282C rescued mutant.
